# Challenges and Limitations in Distributional Cost-Effectiveness Analysis: A Systematic Literature Review

**DOI:** 10.3390/ijerph20010505

**Published:** 2022-12-28

**Authors:** Dirk Steijger, Chandrima Chatterjee, Wim Groot, Milena Pavlova

**Affiliations:** 1 Master’s Program Global Health, Faculty of Health, Medicine and Life Sciences, Maastricht University, P.O. Box 616, 6200 MD Maastricht, The Netherlands; 2 Department of Health Services Research, CAPHRI, Maastricht University Medical Centre, Faculty of Health, Medicine and Life Sciences, Maastricht University, P.O. Box 616, 6200 MD Maastricht, The Netherlands

**Keywords:** equity, distributional cost-effectiveness analysis, health technology assessment

## Abstract

Background: Cost-effectiveness is a tool to maximize health benefits and to improve efficiency in healthcare. However, efficient outcomes are not always the most equitable ones. Distributional cost-effectiveness analysis (DCEA) offers a framework for incorporating equity concerns into cost-effectiveness analysis. Objective: This systematic review aims to outline the challenges and limitations in applying DCEA in healthcare settings. Methods: We searched Medline, Scopus, BASE, APA Psych, and JSTOR databases. We also included Google Scholar. We searched for English-language peer-reviewed academic publications, while books, editorials and commentary papers were excluded. Titles and abstract screening, full-text screening, reference list reviews, and data extraction were performed by the main researcher. Another researcher checked every paper for eligibility. Details, such as study population, disease area, intervention and comparators, costs and health effects, cost-effectiveness findings, equity analysis and effects, and modelling technique, were extracted. Thematic analysis was applied, focusing on challenges, obstacles, and gaps in DCEA. Results: In total, 615 references were identified, of which 18 studies met the inclusion criteria. Most of these studies were published after 2017. DCEA studies were mainly conducted in Europe and Africa and used quality health-adjusted measurements. In the included studies, absolute inequality indices were used more frequently than relative inequality indices. Every stage of the DCEA presented challenges and/or limitations. Conclusion: This review provides an overview of the literature on the DCEA in healthcare as well as the challenges and limitations related to the different steps needed to conduct the analysis. In particular, we found problems with data availability, the relative unfamiliarity of this analysis among policymakers, and challenges in estimating differences among socioeconomic groups.

## 1. Introduction

Publicly funded health systems need to determine which interventions should be purchased and provided for recipients. However, resource allocation decisions are made in the context of unlimited demand for healthcare and finite budgets to satisfy it. Therefore, priorities should be set concerning what could be covered by public healthcare resources and what should be left to private decisions of patients. When designing and prioritizing interventions, healthcare decision makers often focus on improving social efficiency, reducing unfair health inequalities, and improving total population health [1].

The term health inequality refers to differences in the health status of individuals or groups. Any measurable aspect of health that varies across individuals or socially relevant groupings can be called health inequality. In contrast, health inequity is a specific type of health inequality that denotes an unjust difference in health. By one standard definition, allowing inequalities to persist is unfair because they signify preventable and unnecessary differences. In this sense, health inequities are systematic differences in health that could be avoided by reasonable means. The key distinction between the terms health inequality and health inequity is that the former is simply a dimensional description employed whenever unequal quantities exist. At the same time, the latter encompasses a moral judgment that inequality is wrong [2].

Despite considerable attention to the problem of health inequities in the previous decades, striking differences in health still exist between and within countries today. For example, in 2013, the average life expectancy at birth in countries ranged from 46 years in Sierra Leone to 84 in Japan [3]. Within countries, health inequities can be substantial as well. In India, for example, individuals from the poorest quintiles of families are 86% more likely to die at an early age than those from the wealthiest quintile of families, even after accounting for the influence of age, gender, and other factors likely to influence the risk of early death [2]. 

Health equity is, however, not the only policy concern. Policymakers often use economic evaluations to maximize health benefits and to improve efficiency in healthcare. However, both equity and efficiency of a health intervention are essential, although it is rather challenging to achieve both at the same time due to the trade-off between the two and the limited resources and choices that consequently need to be made. Thus, policymakers often need to balance equity and efficiency, and choose between delivering more equitable health outcomes or more efficient health interventions. This is called the equity-efficiency trade-off. Those trade-offs are laden with assumptions and value judgments [4]. Specifically, surveys involving decision makers have shown that they prioritize those who are younger, sicker, or have lower life expectancies. Decision makers are sometimes comfortable foregoing efficiency to ensure equal access to essential healthcare. Yet, efficiency often takes precedence over equity [5].

Policymakers have various economic evaluation tools to make these complex resource allocation choices. One such tool is cost–benefit analysis (CBA), which is grounded in a welfarist theory and generally takes a “societal approach” to value costs and consequences. Whether or not to implement the intervention rests simply on whether the benefits outweigh the costs; all outcomes must be valued in monetary terms [6]. Because of collective financing mechanisms, social welfare is not revealed through the market due to market failures in healthcare. Therefore, individual preferences often need to be proxied by eliciting the value of the consequences of implementing healthcare interventions. This can be achieved, among others, using the willingness to pay (WTP) approach [7]. Another economic evaluation tool is the cost–effectiveness analysis (CEA), which primarily focuses on the efficiency of healthcare interventions and analyses health outcomes relative to costs. The health outcomes are usually measured in quality-adjusted life years (QALYs) [8]. Traditional CEA puts attention on efficiency, although policymakers could consider other principles as well, such as equity. However, these considerations are not always formally incorporated into economic evaluation techniques applied hitherto [5].

Over the recent years, various modified economic evaluation approaches have emerged that aim to include equity dimensions. The recent review by Ward and colleagues [9] groups those methods into equity-based weighting, extended cost–effectiveness analysis (ECEA), distributional cost–effectiveness analysis (DCEA), multi-criteria decision analysis (MCDA), and mathematical programming. Two of those methods, namely equity-based weighting and mathematical programming, require adjusting the incremental cost–effectiveness ratios (ICER) to account for expected equity effects, while MCDA uses scoring/ranking systems for the assessment of the intervention based on a variety of criteria. ECEA and DCEA address equity differently. ECEA broadens the traditional CEA analysis by analyzing the expected changes in financial risk protection as a result of the intervention, while DCEA broadens the traditional CEA by analyzing the expected distribution of intervention costs and QALYs across population groups. Although ECEA and DCEA show some similarities at first glance, they have very different analytical foundations and facilitate a distinct set of decision-making questions. 

For example, ECEA aims to incorporate the equity effects of an intervention on health and financial risk protection to support decisions on extending the coverage of that intervention. It provides information on the equity consequences of the intervention disaggregated across different population strata relevant to the decision (i.e., geography or socioeconomic status) in terms of health gains and household expenditure averted [10]. However, different ECEA studies may use various health outcome measures and other measures of financial risk protection. Therefore, they are not always directly comparable.

DCEA offers an alternative framework for incorporating equity aspects into CEA. Unlike ECEA, DCEA allows for the analysis of multiple distributional variables in addition to the wealth quintile group. DCEA is set within an extra-welfarist framework that considers how health interventions affect the distribution of health. Specifically, it estimates the changes in costs and outcomes resulting from a new intervention as traditional CEA. However, it also estimates the distribution of costs and outcomes across population groups before and after the introduction of the new intervention. Thus, it identifies groups who benefit from the intervention and those who lose out due to the changes [9]. DCEA is therefore used to estimate the net impact of an intervention on overall health and in each population group of interest and to examine the trade-offs between improving overall health and reducing health inequity [11]. DCEA consists of two main stages: (1) modeling the social distribution of health associated with each intervention and (2) evaluating social distributions of health. Thus, DCEA provides additional information about fairness in distributing costs and effects: who gains, who loses, and by how much [1,12]. 

This study aims to identify empirical studies on DCEA in healthcare and outline the challenges and limitations of applying DCEA in healthcare settings. The review helps to create an integrated resource for understanding the methodological aspects of health inequity trade-offs in this economic evaluation approach to help advance health inequity research. Moreover, DCEA is a relatively new method that fills a knowledge gap in the economic evaluation literature. It has been applied in health economic evaluations since approximately 2015 and has been included in broader systematic reviews next to other methods [5]. However, none of the literature reviews in the area of economic evaluation methods have explicitly focused on the DCEA method and have summarized the patterns and challenges of DCEA applications and related equity/efficiency trade-offs conducted.

## 2. Materials and Methods

This systematic review follows the Preferred Reporting Items for Systematic Reviews and Meta-Analysis (PRISMA) guidelines. We used the PRISMA 2020 checklist to ensure that we covered all the items in an evidence-based manner (see Table S1). The review is an integral part of a broader review, for which protocol has been agreed and registered in PROSPERO (protocol number CRD42020202012) prior to the start of this specific review on DCEA.

### 2.1. Search Strategy

We searched Medline (via PubMed), Scopus, BASE, APA Psych, and the JSTOR databases. We chose those databases because they include the most articles on health economics and public health background. Besides those databases, we also searched the Google Scholar database to verify that no relevant paper had been missed. First, we searched the Medline database via PubMed. After that, we searched in the following sequence: Scopus, BASE, APA Psych, JSTOR, and Google Scholar. We searched for English-language literature published on or before our final search, which took place on 1 May 2022. 

We used the following search terms: “distributional costs effectiveness analysis” OR “DCEA” OR “distributional economic evaluation.” These search terms were selected after a thorough iterative process of adding, changing and deleting search terms and search term combinations, based on the initial broader review. All relevant articles found a priori in the broader review, where general terms related to equity were included, appeared in this focused search as well, which confirmed the adequacy of the search strategy. Moreover, this combination of keywords had a minor number of off-topic articles. 

### 2.2. Study Screening and Selection

We included articles if they applied DCEA and if they explored the costs and health outcomes of two or more alternative healthcare interventions. The articles had to be full-text papers. There were no restrictions regarding the target population group, comparison group, and/or outcomes measured. We also included articles that discussed the method of DCEA. We excluded books, systematic or narrative reviews, and editorial and commentary papers. 

The main researcher screened the articles per database based on title and abstract. A second researcher checked the selection. We used the software application EndNote to save and share the articles with all researchers involved in the review. After the initial screening, the main researcher retrieved the full text of all articles assessed as relevant based on title and abstract and checked their eligibility. A second researcher again checked the selection. Finally, the main researcher hand-searched the reference lists of the included and excluded articles (including the reference lists of the excluded review papers). This helped to determine if studies were missed in the original search. Unique titles identified during this final screening step were assessed for eligibility based on the same inclusion criteria. No automation tool was used for identifying duplicates. Each article was compared with the Endnote bibliography to eliminate duplicates. 

### 2.3. Quality Assessment

To critically appraise the eligibility of the papers, we used the Critical Appraisal Skills Program (CASP) checklist. Every paper was assessed individually by this checklist, and assessment results were documented in a Microsoft Excel table. After the first author critically appraised the full-text articles, a second author also critically appraised and assessed each paper individually. The results were compared among the authors to evaluate the risk of bias. There were no significant differences to be found.

### 2.4. Data Extraction and Analysis

After the critical appraisal of the eligibility of the papers, one author read the full-text articles and extracted the following from the included articles: article details (authors and date of publication), geography and country, study population, disease area, intervention and comparators, intervention costs, measure of health effect, base-case cost–effectiveness findings, and equity analysis results, modeling technique and equity effect. We also extracted information about the challenges and limitations of DCEA mentioned in articles where the method was discussed. Thus, a thematic analysis was applied based on the above themes, which were pre-defined given the review objective. We used Microsoft Excel to create an extraction matrix and to save the extracted results. Weekly meetings were held during the period of data extraction and analysis to discuss the results and ensure the review quality. The extracted results were synthesized and presented narratively. Tables were used to illustrate the results.

## 3. Results

In total, 615 citations were identified using the search strategy. PubMed gave 28 citations, Scopus gave 239 citations, BASE gave four citations, APA Psych gave 27 citations, and Google Scholar gave 317 citations. JSTOR gave no results. After screening all the titles and abstracts, 64 records were sought for retrieval. Twenty-one records were not retrieved because the full text was not available, the full-text article was not written in English, or the full-text article could not be found. After this step, 41 articles were critically appraised for eligibility. 

During the critical appraisal, 24 full-text articles were excluded from the search due to irrelevance. The most common reason for exclusion was that the full-text article was not about DCEA. For example, 12 articles mentioned DCEA in the abstract or title but only mentioned the abbreviation in the discussion section, whereas the article referred to other methods for CEA. The second most common reason for exclusion was that the full text was no longer available. This happened only for articles that were found through Google Scholar. We searched those articles in other databases. However, we were unable to find seven articles. The last reason for exclusion was that the study was duplicated from another study but with a slightly different abstract and/or introduction section. The five articles excluded for this reason also came from Google Scholar only. 

**Figure 1:**
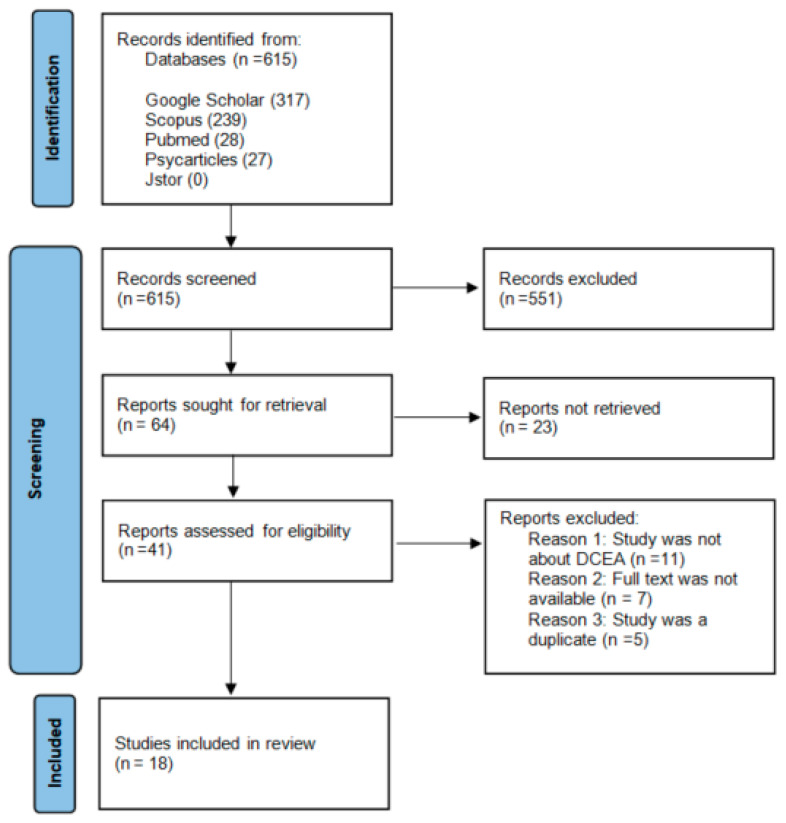
PRISMA flowchart; search and selection process.

Eventually, 18 studies were included in this review. The search process is presented in the PRISMA diagram in Figure 1. In Tables S2 and S3, the details of the articles reviewed can be found. All papers reviewed are presented in Table S2, while Table S3 only lists papers that present an empirical application of the DCEA method.

### 3.1. Study Characteristics

The key characteristics of the DCEA studies reviewed are summarized in Table 1. Most of the studies (88.9%) were published after 2017. Around half of the studies were conducted in Africa (Tanzania, Ethiopia, and Malawi) and Europe (the United Kingdom). Approximately 27.8% of the studies focused on multiple diseases. Diseases included cancer (adenocarcinoma and cervical cancer), cardiovascular disease, and infectious diseases (pneumonia and HIV). Both screening and treatment interventions were evaluated in three studies. An immunization or infectious intervention was assessed once. In one-third of the studies, the general population or adults were the main populations of focus, followed by children. In total, 66.7% (12 out of 18) were empirical studies, and 6 (33.3%) papers were descriptive papers about the DCEA method. The main subjects of these papers were the impact of socioeconomic differences and variations in economic evaluations, methods to promote equity in DCEA, and an early overview of the academic literature around DCEA.

As indicated in Table 2, slightly more than half of the included studies presented an economic evaluation with a societal perspective. Four (22.2%) studies presented economic evaluation with a healthcare perspective. In addition, four (22.2%) studies did not mention the perspective of analysis because they described the mathematical process of the analysis in general.

Treatment or intervention costs were the most frequent cost issue (66.7%). Provider costs were the second most examined cost issue. However, seven studies that examined treatment and intervention costs also studied the opportunity costs of other options. Two studies examined opportunity costs only. In total, two (11.1%) studies focused on provider and patient costs. 

The quality-, disability- or health-adjusted measurements (QALY, DALY, HALE) were the most frequently measured health outcomes (77.8%). Other health outcomes such as deaths averted and life expectancy were used in two studies (11.1%), separately. 

Regarding the types of simulation that have been used for the analysis, a Markov model was the most used (33.3%). In addition, specific rates (age, region, and gender) were obtained from surveys and national statistics, and the treatment effects were collected from systematic reviews. In total, five (27.8%) papers did not mention any type of simulation, and four (22.2%) papers used a dynamic simulation model, which used data from systematic reviews and other scientific literature primarily.

The most used equity indices were the equally distributed equivalent (44.4%) and the Atkinson index (33.3%). In addition, the Kolm index (16.7%), the Gini index (22.2%), the slope index of inequality (22.2%), the relative index of inequality (11.1%), the Theil index (5.6%), and the index of disparity (5.6%), were also used in the included studies. Six of the included studies used more than one equity measurement.

### 3.2. Challenges and Limitations in the Application of the DCEA Method

According to the DCEA framework of Asaria et al. [12], conducting a DCEA has two main stages. We focus below on the challenges and limitations reported in each stage.

#### 3.2.1. Stage 1: Modelling the Social Distribution of Health

The first DCEA stage is used to model the social distribution of health associated with each intervention. This is accomplished by first estimating the baseline health distribution. Next, changes to this baseline distribution due to the health interventions are modeled. After that, adjustments are made for social value judgments about fair and unfair sources of inequality.

- Estimating baseline social distribution of health

DCEA starts with describing the baseline distribution of health, considering variation in the length and health-related quality of life. This baseline health distribution should explain the variation in health among multiple different subgroups in the population as defined by the relevant population characteristics [13]. 

The most challenging aspect of estimating the baseline health distribution is to identify reliable and vital health statistics. Many researchers use reliable health data from established institutes when conducting a DCEA in high-income countries. At the same time, it is more challenging to complete a DCEA in low-income countries. For example, studies in low-income countries mention the lack of a vital registration system for adult mortality, and therefore, data on life expectancy from the WHO need to be used. In some cases, however, there are no reliable data, even in high-income countries. 

Moreover, health statistics in low-income countries are often obtained from surveys. These surveys are usually not annually or bi-annually conducted, which lead to biased estimations when data are not gathered periodically and/or data for certain periods are missing. Furthermore, the data collected in those surveys do not necessarily match with data from other surveys available in the country, which makes it difficult to combine datasets in a joint analysis. It is plausible that some data are collected during the high season of a communicable disease, or patients with non-communicable diseases might have diverse comorbidities and experience a variety of symptoms not directly related to the health issues being studied [14,15,16]. 

Furthermore, health data are hard to obtain in low-income countries, and there is also a gap in data on population characteristics, primarily on regional and rural population density data. To fill this gap, studies resort to making assumptions about population characteristics by comparing the research population with a neighboring country [8,14,15,17]. 

A challenging aspect is also the estimation of differences between socioeconomic groups. Often, health data from institutions in high- and middle-income countries use statistical variables such as age, sex, and region-specific data. However, a lack of reliable data regarding these variables often appears when estimating health distribution by incorporating socioeconomic variables. Again, this occurs even more often in low-income countries [14,18].

- Modeling changes in the baseline distribution due to the compared interventions

After the estimation of the baseline health distribution, it is necessary to examine how changes in the baseline health distribution could be modeled by comparing and applying health interventions to the study population. The purpose is to evaluate changes in the baseline health distribution that could be attributed to alternative interventions. In particular, it needs to be decided how the costs and effects of the intervention differ between the relevant subgroups. The modeling of changes to the baseline distribution by health interventions also presents some challenges and limitations.

First of all, as mentioned above, a challenging aspect is collecting reliable and robust data. It is sometimes difficult to incorporate evidence on the differential uptake and to consider the implications of evidence on differential effectiveness between different groups [16]. These groups could differ from socioeconomic groups to race or ethnicity groups. If this is not accounted for, it could lead to underestimation or overestimation of the true extent of differential effectiveness [19]. Information describing differences in healthcare utilization for each disease area between equity-relevant groups accounts for differences in uptake. However, simply using the distribution of utilization to allocate the health effect assumes that each particular episode of care generates the same health outcomes, regardless of the social characteristics of the recipient. For example, in the cancer disease area, using the socioeconomic distribution of surgical removal of tumors to describe the distribution of the health benefits from surgery would assume that every individual achieves equal benefits from undergoing surgery regardless of socioeconomic status [18]. 

Furthermore, making assumptions about the intervention uptake probabilities can also be challenging. Because using existing patterns of utilization may be biased if a new technology is expected to change uptake patterns across social groups. If the provision of a new treatment is likely to increase uptake in the most disadvantaged groups, then current utilization will underestimate the benefits to health inequality. However, if data on expected uptake patterns are available, they can be used to adjust or replace the healthcare utilization distributions used to allocate health benefits [8].

- Adjusting for social value judgments about fair and unfair sources of inequality

The first stage of the DCEA is completed by adjusting for social value judgments about fair and unfair sources of inequality. Thus, the distributions of health estimated represent all population health variations. However, some variations in health may be deemed “fair” or at least “not unfair” because, for example, it is due to individual choice or unavoidable bad luck. In such cases, the health distributions should first be adjusted only to include health variation deemed “unfair” before measuring the level of inequality. Social value judgments need to be made about whether the health variation associated with each population characteristic is fair [12,13]. 

An issue requiring value judgment could be the choice of the health outcome metric and how far this may indirectly discriminate against disadvantaged groups. There is significant ethics and economics literature on how outcome metrics (HALE, QALE, DALE, etc.), commonly used in health economic evaluation, may implicitly discriminate against preventing mortality or other health effects among relatively unhealthy population groups such as the poor, the elderly and the disabled. This indirect discrimination could occur because relatively few years of healthy life are gained from averting the death of a somewhat unhealthy individual [15]. It is essential for policymakers and health economic researchers to consider this ethical issue and how far it may or may not be relevant to the case at hand. Where the issue is considered relevant by stakeholders, analysts can address it using sensitivity analysis based on simple binary outcome metrics, such as mortality or cases of disease averted, which do not indirectly discriminate in this way.

#### 3.2.2. Stage 2: Evaluation of Social Distribution of Health

The second stage is to evaluate social distributions of health. First, it is necessary to use the estimated distributions to quantify the change in total population health and unfair health inequality due to each intervention. Afterward, researchers should rank the interventions based on dominance criteria. Finally, the research needs to analyze the trade-offs between improving population health and reducing unfair health inequality [13].

- Quantifying health inequality due to intervention and ranking the interventions

As equity is often defined in terms of fairness between social groups, measuring it requires information on demographic and socioeconomic variables. Incorporating more variables allows for a more nuanced perspective on inequality, as it affects specific societal subgroups [13]. The level of inequality can then be estimated using a wide range of metrics. Frequently used tools include gap measures, regression-based measures, concentration curves, measurements incorporating inequality aversion, and inequality measurements with health-related social welfare functions. They vary considerably in their sophistication [18].

Here, we characterize the distributions regarding the twin policy goals of improving total health and reducing health inequality. One helpful piece of information for decision makers produced at this step of the analysis is the size of the health opportunity cost of choosing an intervention that reduces health inequality [12]. However, the analysis can also go further than that by providing information about the size of the reduction in health inequality in terms of the difference in one or more suitable inequality indices between the intervention and a comparator. Commonly used methods to measure inequality can be broadly grouped into those measuring relative inequality, absolute inequality, and health poverty or shortfall from a reference value. If there is no clear choice of inequality measure; it may be preferable to calculate a range of alternative measures [12,16]. 

The change in total population health and unfair health inequality due to each intervention is used to rank the interventions (dominance rule applied). It is also possible to use a relative inequality index to assess inequalities and rank the interventions. Relative inequality measures show proportional differences in health among subgroups [18]. An issue requiring quantifying unfair health inequality could be the choice of which inequality measures to adopt. This may depend upon the availability of data and the decision-making context. For example, simple gap statistics and concentration curves can be simpler to compute and are more easily interpretable by a non-technical audience.

Finally, it could be challenging to measure inequality between social-economic groups when the number of residents in these groups is skewed [20]. For example, when there is a small number of people in the least deprived group, comparisons should only be made between the second least disadvantaged group and the most deprived group. The possibility of merging the least deprived group with the second least deprived group was not discussed in the papers included.

- Analyzing the trade-offs between improving health and reducing inequality

In cases where the dominance rule does not provide a complete ordering of strategies, different social value judgments are required to assess trade-offs between improving total population health and reducing unfair health inequality. The key additional social value judgments that need to be made are related to the choice of inequality measures underpinning social welfare and the level of inequality aversion [21]. Studies often use high and low levels of inequality aversion. They then assess the sensitivity of their decisions across a range of inequality aversion levels to identify the thresholds at which each strategy would be preferred.

Finally, an extensive sensitivity analysis should be performed. Alternative assumptions about the distribution of the intervention outcomes, the baseline health distribution, and the inequality aversion level need to be tested. For example, an assumption could be: the direct health benefit of each person receiving intervention is the same regardless of socioeconomic status, i.e., we assume equal efficacy of interventions across socioeconomic groups [21]. Information obtained about increasing total health improvements and reductions in health inequality are presented in the studies. This information enables them to reimburse interventions that have a positive impact on the net health outcomes as well as to reduce health inequality. 

Trade-offs could occur when a DCEA study compares interventions that include a full range of scale-up scenarios. Potential constraints on each intervention scale-up level should be considered because inequalities across different groups affect the utilization. If those practical constraints are included in the analyses, this could lead to a skewed inequality measurement, which results in a biased policy solution. Currently, a DCEA is often the first of its kind in the area of a given disease in a specific country or region. Thus, in many cases, no other results are available to compare the study findings. A biased discussion-making process could occur if decisions depend on only one reliable study.

## 4. Discussion

This is the first study that systematically identifies and summarizes DCEA studies in healthcare and outlines the challenges and limitations in applying DCEA in healthcare. The review identified 18 relevant empirical studies. The majority of these studies were published after 2017 and focused on a variety of diseases, intervention types, and populations. We crystallized the characteristics of the design and health effects. We found that the DCEA studies were conducted in high- and low-income countries. DCEA studies in high-income countries were almost always conducted in the United Kingdom. As shown by the review results, the main challenges in the process of a DCEA were the lack of reliable data and estimating differences between socioeconomic differs groups.

The first DCEA was conducted in 2015, and then, the literature increased during the subsequent years, with a peak in published articles in 2020 and 2021. None of the DCEA literature in these later years was related to COVID-19. This is somewhat unexpected because governments raised their public health expenditure to control the pandemic. Economic evaluation tools incorporating equity, such as DCEA, might have been insightful for their decisions since these extra funds aimed to improve health and reduce health inequalities [22]. As we have described, a DCEA is ideally suited for this because of the societal perspective of the analysis. However, policymakers stuck to the more conventional CEA that prevailed [23,24,25]. 

While we found that DCEA was primarily applied in research in the United Kingdom, other cost-equity informative cost-effectiveness analyses, such as equity-based weighting, MCDA and ECEA, have been used to address equity effects in a much more diverse set of countries [5,9]. The limited application of DCEA is however attributed to its recent emergence and not to its nature. The review by Ward and colleagues [9] argues that DCEA is the only method thus far that shows no major methodology concerns except that it is still unfamiliar and requires additional data collection. Still, data availability is reported to be less of a problem in DCEA than, for example, in the case of equity-based weighting and MCDA, which require preference elicitation to decide on equity weights and relevant attributes, respectively. In addition, it is argued that DCEA, similar to ECEA and MCDA, is superior to equity-based weighting and mathematical programming when changes in outcome inequality need to be analyzed. DCEA is also relevant to all types of health systems, and this is not the case for ECEA, which is less appealing in countries with good financial protection in healthcare. 

The challenge that the DCEA method is not yet sufficiently known and accepted among policymakers could be overcome by increasing the research literature on this topic to confirm the rigorousness of the method. In addition, DCEA should be given more attention during the curricula in medical education for future (public) health experts. Currently, the focus is mainly on traditional CEA [26,27].

Another critical challenge in DCEA is the availability of data, even though it is less problematic compared to other methods, as discussed above. Specifically, data required for the DCEA can often be obtained from existing sources (e.g., datasets or surveys). However, those sources should also be consistent and reliable. In DCEA, data quality is especially essential for accurately identifying inequalities within the study population and the changes in inequalities after the intervention. Without adequate data, inequities remain unseen and perhaps unaddressed [28,29]. Although CEA in which results are collected through a randomized controlled trial is less affected by this issue, it is still a recognized problem for every CEA as well [30,31,32,33]. Better-informed decisions can be made before implementing an intervention with access to real-time data [1]. Specifically, for conducting a DCEA, the problem could be overcome by improving the availability and quality of critical (health) data sources. 

A commentary on this topic by Meunier and colleagues [32] suggests that governments and HTA agencies need to define which equity concerns are relevant in the realm of CEA and how to best measure them. It is also important to link databases from different research fields and enrich existing registries (real-world data) to ensure relevant input for future DCEA studies. Collaboration between healthcare stakeholders will be important to ensure the quality of these joint datasets and registers and adequate data protection. It is also important to support and stimulate new DCEA applications. In this regard, observational studies can be especially useful in enhancing our understanding of health inequities and heterogeneity in treatment effectiveness. In addition, longitudinal studies are needed to evidence the trends in inequities over time. Countries should also increase their database capacity to address fundamental health statistics needs. In some low-income countries, this also means improved registration of births and deaths per region, thus strengthening the countries’ health information systems. Countries should also assess their health information systems by the World Health Organization standards to examine flaws in their health systems [34]. 

Another common challenge in DCEA is how to estimate differences between socioeconomic groups. Simply focusing on a group’s average levels of health is not sufficient. Even within the same socioeconomic group, an intervention can affect individuals differently. Some DCEA studies emphasize that the solution is to move toward the measurement of the distribution across individuals’ health [35,36]. However, this will not solve the challenge of adequately estimating the health differences because the individual health indicators are as good and accurate as the data used. Furthermore, the problem of comparability of individual health data also exists. Standardized data collection is proposed as a more adequate solution [37], although it is almost impossible to fully standardize data collection, which is an important limitation. 

We also need to recognize that DCEA possesses limitations typical for traditional CEA as well. For example, although DCEA incorporates variations in health outcomes across population subgroups due to the intervention, not all inequalities are considered. In particular, there are also variations in the quality of healthcare received as well as variations in benefits coverage. These and other service-related differences are not captured in DCEA. This weakness is typical of all economic evaluation studies. 

This systematic review has some limitations. First, we searched several databases to improve the comprehensiveness of the search, but we may still have missed articles listed in other databases and articles published in a language other than English, as well as articles in the process of publication. Thus, bias related to our search strategy cannot be completely excluded. Second, although 90% of included studies met the quality criteria in the CASP checklist, the quality assessment did not explicitly focus on the DCEA but only on how the studies are reported. This suggests the need for a DCEA quality checklist in the future. Third, we made an attempt to reduce the selection bias by having a second researcher check the selection process. However, selection bias cannot be completely excluded since the second researcher did not directly check the extraction process. Still, we had a weekly discussion on data extraction and analysis. We also acknowledge that it is not possible to make generalizations given the small number of studies. We, therefore, cannot recommend specific methodology improvements of DCEA, and we cannot identify methodology differences in DCEA arising from the specificities of the interventions, outcomes and/or equity indicators being studied. These points could be the subject of future reviews when more empirical applications of the DCEA method become available. 

## 5. Conclusions

This study outlined the DCEA challenges and limitations when applied to healthcare. This study is useful for researchers and policymakers because we described and elaborated on the key issues in each DCEA step. This is the first review in the field of DCEA with this perspective. It can help to understand the methodological issues the DCEA method poses at every step to help advance health inequity research. 

We identified several essential challenges and limitations. In particular, the availability of data, the relative unfamiliarity of this analysis among policymakers and other stakeholders, and the issue of how to estimate differences between socioeconomic groups. There is a need to standardize the inclusion of equity in DCEA, and in particular, what and how to measure it. This process can best be guided by governments and HTA agencies to ensure the usefulness of DCEA for decision making, which will enhance the trust in this new method. Future research should focus on the specificities of applying this method in a low- or middle- or high-income country. This will shed light on challenges and limitations that have not been captured in the publications we reviewed. 

Based on the literature, we also indicated five directions for improving data availability for future DCEA studies: linking databases from different research fields to offer easier access to equity-related data; enriching existing registries (real-world data) to provide input for future studies; supporting and stimulating new DCEA applications, including observational and longitudinal studies; expanding database capacity, especially in low-income countries; and overall strengthening the national information systems according to WHO standards. 

To explore further the challenges and limitations of DCEA, in-depth interviews and focus group discussions could be used in future studies. Such studies could highlight how to improve the method and its application, taking into account the specific country and setting.

## Figures and Tables

**Table 1:** General characteristics of the included studies (N = 18).

	Number	Percent (%) *	Article Number in Table S2
Year of publication			
Before 2017	2	11.1	1,2
2017 or later	16	88.9	3–18
Geographical area			
Asia	1	5.6	4
Africa	4	22.2	2,3,7,16
Europe	5	27.8	1,6,8,9,12
North America	1	5.6	17
South America	1	5.6	15
Others	6	33.3	5,10,11,13,14,18
Disease area			
Cancer	2	11.1	1,4
Cardiovascular disease	3	16.7	2,8,16
Infectious disease	2	11.1	3,17
Multiple diseases	5	27.8	6,7,9,12,15
Others	6	33.3	5,10,11,13,14,18
Intervention type			
Screening	3	16.7	1,4,8
Immunization	1	5.6	3
Healthcare treatment	3	16.7	2,16,17
Behavior intervention	1	5.6	9
Multiple interventions	3	16.7	7,12,15
Others	7	38.9	5,6,10,11,13,14,18
Study population			
Adults	3	16.7	1,2,8
Children	2	11.1	3,16
Women	1	5.6	4
General population	4	22.2	7,9,12,15
Patients	1	5.6	6
Others	6	33.3	5,10,11,13,14,18

* Share of publications reviewed; a publication can be classified into multiple categories.

**Table 2:** Design characteristics of the included studies (N = 18).

	Number	Percent (%) *	Article Number in Table S2
Perspective of analysis			
Healthcare	4	22.2	1,2,4,15
Societal	10	55.6	3,5–9,12,13,16,17
Not stated	4	22.2	10,11,14,18
Type of costs			
Treatment/intervention costs	12	66.7	1,3,4,6–9,12,15-18
Provider and patient costs	2	11.1	2,16
Opportunity cost	9	50.0	3,5–9,12,13,15
Not stated	3	16.7	10,11,14
Measures of health benefits			
Quality-, disability-or health-adjusted life years	14	77.8	1,3–9,11–13,15,17,18
Deaths averted/mortality rate	2	11.1	3,16
Life expectancy	2	11.1	2,16
Not stated	2	11.1	10,14
Type of simulation			
Markov model	6	33.3	2,4,9,13,15,16
Dynamic simulation	4	22.2	1,3,8,17
Decision model	1	5.6	12
Aggregated effectiveness model	2	11.1	6,7
Not stated	5	27.8	5,10,11,14,18
Equity effects			
Atkinson index	6	33.3	1,4,7,10,12,13
Equally distributed equivalent	8	44.4	3,5–7,9,12,13,15
Gini index	4	22.2	1,2,10,16
Index of disparity	1	5.6	17
Kolm index	3	16.7	1,10,12
Relative index of inequality	2	11.1	5,11
Slope index of inequality	4	22.2	1,5,8,11
Theil index	1	5.6	17
Not stated	2	11.1	14,18

* Share of publications reviewed; a publication can be classified into multiple categories.

## Data Availability

The dataset (list of included articles) supporting the conclusions of this article are included within the tables in this article and in the supplementary files.

## Supplementary Materials

The following supporting information can be downloaded at: https://www.mdpi.com/article/10.3390/ijerph20010505/s1, Table S1: Prisma checklist 2020 [38], Table S2: Articles included in the systematic review, Table S3: Study design and effect characteristics.
