# Supplementary material for: Challenges and Limitations in Distributional Cost-Effectiveness Analysis: A Systematic Literature Review
Source: Int J Environ Res Public Health. 2022 Dec 28;20(1):505. doi: 10.3390/ijerph20010505 (PMC9819735; doi:10.3390/ijerph20010505)
Supplement: Supplementary file 1 [file ijerph-20-00505-s001.zip › ijerph-2045908-supplementary.pdf]

Table S1: Prisma Checklist 2020

PRISMA 2020 Checklist

Section and Topic	Item #	Checklist item	Location where item is reported
TITLE			
Title	1	Identify the report as a systematic review.	Front page
ABSTRACT			
Abstract	2	See the PRISMA 2020 for Abstracts checklist.	Abstract section
INTRODUCTION			
Rationale	3	Describe the rationale for the review in the context of existing knowledge.	Introduction
Objectives	4	Provide an explicit statement of the objective(s) or question(s) the review addresses.	Introduction
METHODS			
Eligibility criteria	5	Specify the inclusion and exclusion criteria for the review and how studies were grouped for the syntheses.	Methods
Information sources	6	Specify all databases, registers, websites, organisations, reference lists and other sources searched or consulted to identify studies. Specify the date when each source was last searched or consulted.	Methods
Search strategy	7	Present the full search strategies for all databases, registers and websites, including any filters and limits used.	Methods
Selection process	8	Specify the methods used to decide whether a study met the inclusion criteria of the review, including how many reviewers screened each record and each report retrieved, whether they worked independently, and if applicable, details of automation tools used in the process.	Methods
Data collection process	9	Specify the methods used to collect data from reports, including how many reviewers collected data from each report, whether they worked independently, any processes for obtaining or confirming data from study investigators, and if applicable, details of automation tools used in the process.	Methods
Data items	10a	List and define all outcomes for which data were sought. Specify whether all results that were compatible with each outcome domain in each study were sought (e.g. for all measures, time points, analyses), and if not, the methods used to decide which results to collect.	Methods
	10b	List and define all other variables for which data were sought (e.g. participant and intervention characteristics, funding sources). Describe any assumptions made about any missing or unclear information.	Methods
Study risk of bias assessment	11	Specify the methods used to assess risk of bias in the included studies, including details of the tool(s) used, how many reviewers assessed each study and whether they worked independently, and if applicable, details of automation tools used in the process.	Methods
Effect measures	12	Specify for each outcome the effect measure(s) (e.g. risk ratio, mean difference) used in the synthesis or presentation of results.	NA
Synthesis methods	13a	Describe the processes used to decide which studies were eligible for each synthesis (e.g. tabulating the study intervention characteristics and comparing against the planned groups for each synthesis (item #5)).	NA
	13b	Describe any methods required to prepare the data for presentation or synthesis, such as handling of missing summary statistics, or data conversions.	NA
	13c	Describe any methods used to tabulate or visually display results of individual studies and syntheses.	Methods
	13d	Describe any methods used to synthesize results and provide a rationale for the choice(s). If meta-analysis was performed, describe the model(s), method(s) to identify the presence and extent of statistical heterogeneity, and software package(s) used.	NA
	13e	Describe any methods used to explore possible causes of heterogeneity among study results (e.g. subgroup analysis, meta-regression).	NA
	13f	Describe any sensitivity analyses conducted to assess robustness of the synthesized results.	NA

Section and Topic	Item #	Checklist item	Location where item is reported
Reporting bias assessment	14	Describe any methods used to assess risk of bias due to missing results in a synthesis (arising from reporting biases).	Methods
Certainty assessment	15	Describe any methods used to assess certainty (or confidence) in the body of evidence for an outcome.	Methods
RESULTS			
Study selection	16a	Describe the results of the search and selection process, from the number of records identified in the search to the number of studies included in the review, ideally using a flow diagram.	Results
	16b	Cite studies that might appear to meet the inclusion criteria, but which were excluded, and explain why they were excluded.	Results
Study characteristics	17	Cite each included study and present its characteristics.	Results
Risk of bias in studies	18	Present assessments of risk of bias for each included study.	NA
Results of individual studies	19	For all outcomes, present, for each study: (a) summary statistics for each group (where appropriate) and (b) an effect estimate and its precision (e.g. confidence/credible interval), ideally using structured tables or plots.	NA
Results of syntheses	20a	For each synthesis, briefly summarise the characteristics and risk of bias among contributing studies.	Appendix C
	20b	Present results of all statistical syntheses conducted. If meta-analysis was done, present for each the summary estimate and its precision (e.g. confidence/credible interval) and measures of statistical heterogeneity. If comparing groups, describe the direction of the effect.	NA
	20c	Present results of all investigations of possible causes of heterogeneity among study results.	NA
	20d	Present results of all sensitivity analyses conducted to assess the robustness of the synthesized results.	NA
Reporting biases	21	Present assessments of risk of bias due to missing results (arising from reporting biases) for each synthesis assessed.	NA
Certainty of evidence	22	Present assessments of certainty (or confidence) in the body of evidence for each outcome assessed.	NA
DISCUSSION			
Discussion	23a	Provide a general interpretation of the results in the context of other evidence.	Discussion
	23b	Discuss any limitations of the evidence included in the review.	Discussion
	23c	Discuss any limitations of the review processes used.	Discussion
	23d	Discuss implications of the results for practice, policy, and future research.	Conclusion
OTHER INFORMATION			
Registration and protocol	24a	Provide registration information for the review, including register name and registration number, or state that the review was not registered.	NA
	24b	Indicate where the review protocol can be accessed, or state that a protocol was not prepared.	NA
	24c	Describe and explain any amendments to information provided at registration or in the protocol.	NA
Support	25	Describe sources of financial or non-financial support for the review, and the role of the funders or sponsors in the review.	Declaration section
Competing interests	26	Declare any competing interests of review authors.	Declaration section
Availability of data, code and other materials	27	Report which of the following are publicly available and where they can be found: template data collection forms; data extracted from included studies; data used for all analyses; analytic code; any other materials used in the review.	NA

From: Page MJ, McKenzie JE, Bossuyt PM, Boutron I, Hoffmann TC, Mulrow CD, et al. The PRISMA 2020 statement: an updated guideline for reporting systematic reviews. BMJ 2021;372:n71. doi: 10.1136/bmj.n71

For more information, visit: http://www.prisma-statement.org/

Table S2: Articles included in the systematic review

	Authors	Article title	Publication details
**1.**	M. Asaria, S. Griffin, R. Cookson, S. Whyte and P. Tappenden	Distributional cost-effectiveness analysis of health care programmes--a methodological case study of the UK bowel cancer screening programme.	Health Economics 2015 Jun;24(6):742-54.
**2.**	F.N. Ngalesoni, G.M. Ruhago, A.T. Mori, B. Robberstad and O.F. Norheim	Equity impact analysis of medical approaches to cardiovascular diseases prevention in Tanzania.	Social Science & Medicine 2016 Dec;170:208-217.
**3.**	B.R. Dawkins, A.J. Mirelman, M. Asaria, K.A. Johansson and R.A. Cookson	Distributional cost-effectiveness analysis in low- and middle-income countries: illustrative example of rotavirus vaccination in Ethiopia.	Health Policy and Planning 2018 Apr 1;33(3):456-463.
**4.**	T.H. Lee, W. Kim, J. Shin, E.C. Park, S. Park and T.H. Kim	Strategic distributional cost-effectiveness analysis for improving national cancer screening uptake in cervical cancer: a focus on regional inequality in South Korea.	Cancer Research and Treatment 2018 Jan;50(1):212-221.
**5.**	S. Griffin, J. Love-Koh, B. Pennington and L. Owen	Evaluation of intervention impact on health inequality for resource allocation.	Medical Decision Making 2019 Apr;39(3):171-182.
**6.**	J. Love-Koh, R. Cookson, N. Gutacker, T. Patton and S. Griffin	Aggregate distributional cost-effectiveness analysis of health technologies.	Value in Health 2019 May;22(5):518-526.
**7.**	M. Arnold, D. Nkhoma and S. Griffin	Distributional impact of the Malawian essential health package.	Health Policy and Planning 2020 Jul 1;35(6):646-656.
**8.**	B. Collins, C. Kypridemos, R. Cookson, P. Parvulescu, P. McHale, M. Guzman-Castillo et al.	Universal or targeted cardiovascular screening? Modelling study using a sector-specific distributional cost effectiveness analysis.	Preventive Medicine 2020 Jan;130:105879.
**9.**	S. Griffin, S. Walker and M. Sculpher	Distributional cost effectiveness analysis of west Yorkshire low emission zone policies.	Health Economics 2020 May;29(5):567-579.
**10.**	J. Love-Koh, S. Griffin, E. Kataika, P. Revill, S. Sibandze and S. Walker	Methods to promote equity in health resource allocation in low- and middle-income countries: an overview.	Globalization & Health 2020 Jan 13;16(1):6.
**11.**	J. Love-Koh, R. Cookson, K. Claxton and S. Griffin	Estimating social variation in the health effects of changes in health care expenditure.	Medical Decision Making 2020 Feb;40(2):170-182.
**12.**	J. Love-Koh, B. Pennington, L. Owen, M. Taylor and S. Griffin	How health inequalities accumulate and combine to affect treatment value: a distributional cost-effectiveness analysis of smoking cessation interventions.	Social Science & Medicine 2020 Nov;265:113339.
**13.**	F. Yang, C. Agnus, A. Duarte, D. Gillespie, S. Walker and S. Griffin	Impact of socioeconomic differences on distributional cost-effectiveness analysis.	Medical Decision Making 2020 Jul;40(5):606-618.

**14.**	R. Cookson. S.Griffin, O.F. Norheim, A.J. Culyer and K. Chalkidou	Distributional cost-effectiveness analysis comes of age.	Value in Health 2021 Jan;24(1):118-120.
**15.**	J. Love-Koh, A. Mirelman and M. Suhrcke	Equity and economic evaluation of system-level health interventions: a case study of brazil's family health program.	Health Policy and Planning 2021 Apr 21;36(3):229-238.
**16.**	M. Olsen, O.F. Norheim and S.T. Memirie	Reducing regional health inequality: a sub-national distributional cost-effectiveness analysis of community-based treatment of childhood pneumonia in Ethiopia.	International Journal for Equity in Health 2021 Jan 6;20(1):9.
**17.**	A.M.L. Quan, C. Mah, E. Krebs, X. Zang, S. Chen, K. Althoff et al.	Improving health equity and ending the HIV epidemic in the USA: a distributional cost-effectiveness analysis in six cities.	The Lancet HIV 2021 Sep;8(9):e581-e590.
**18.**	J.P. Jansen, T.A. Trikalinos and K.A. Philips	Assessments of the value of new interventions should include health equity impact.	Pharmacoeconomics 2022 May;40(5):489-495.

Table S3: Study design and effect characteristics

Study identifier	Geography and population	Disease area	Intervention	Costs	Health effects	Equity effects
Asaria et al., 2015	Adults age 30 or above in the United Kingdom	Adenocarcinoma (screening)	1) No intervention 2) standard screening 3) targeted reminder 4) universal reminder	Costs of screening, diagnostic and treatment costs	QALYs<br>ICER (GBP per QALY)	Relative (Atkinson, Gini) and absolute (Kolm , slope index of inequality)
Ngalesone et al., 2016	Adults age 40 or above who are at CVD risk in Tanzania	Cardiovascular disease	1) No intervention, 2) ESC CVD prevention 3) WHO CVD prevention, 4) Differentiated risk threshold approach	Healthcare provider and patient costs	Life expectancy<br><br>ICER (USD per life year)	Gini index
Dawkins et al., 2018	Children under 5 years of age in Ethiopia	Rotavirus	1) No intervention 2) Standard vaccination program 3) Pro poor vaccination program	Vaccine costs + delivering costs and opportunity costs	Mortality and HALYs	EDE health
Lee et al., 2018	Females age 20 or above without a history of cervical cancer in South Korea	Cervical cancer	1) Biennial PSC to all target populations. 2) Biennial PSC to all target populations + strong screening recommendation to target regions. 3) Regular universal PSC recommendation strategy for all target populations. 4) Strong universal PSC recommendation strategy	Direct and indirect costs of screening and treatment.	QALYs<br><br>ICER (KRW per QALY)	Atkinson ICER
Love-Koh et al., 2019	United Kingdom	Wide range of diseases	27 single technology appraisals	Treatment, intervention costs and opportunity costs	QALYs	QALY valuation of change in inequality by comparing the incremental QALYs to the incremental EDE
Arnold et al., 2020	Population of Malawi	Wide range of diseases	51 interventions from the Malawian Essential Health package	Intervention costs and opportunity costs	DALYs	EDE HALE and Atkinson index
Collins et al., 2020	Population with age 30 to 84 years from Liverpool, England	Cardiovascular disease (screening)	1) No CVD screening 2) current screening intervention 3) Increased screening intervention 4) Universal plus targeted screening intervention with	Intervention costs and opportunity costs	QALYs	Reduction in slope index of inequality of rates of incremental net

			top-up delivery to the most deprived fifth.			health benefit per 100,000 person-years.
Griffin et al., 2020	Population from West Yorkshire Yorkshire, England	1)Coronary heart disease 2) chronic bronchitis 3) asthma 4) low weight births 5) preterm births 6) all-cause mortality	17 alternative transport emission reduction strategies compared by no intervention	Intervention costs, social care opportunity costs and individual opportunity costs	QALE	EDE QALE
Love-Koh et al., 2020	Smoking population from in England	Smoking related illnesses	21 behavioural and pharmacological smoking cessation interventions	Health opportunity costs	QALYs	Absolute (Kolm) and relative (Atkinson) EDE health
Love-Koh et al., 2021	60% of the population in Brazil	Wide range of diseases	1) no intervention 2) primary care system level intervention (PSF)	Intervention and opportunity costs	DALYs	EDE DALY
Olsen et al., 2021	Children under 5 years of age in Ethiopia	Pneumonia	1) Baseline sub-national coverage of community based treatment of childhood pneumonia (CCM) 2) scaling up coverage of CCM to 90% coverage in Ethiopia's 11 major regions.	Treatment costs from a providers perspective (divided into patient care costs and overhead costs)	Under 5 mortality rate and average life expectancy at birth	Gini index
Quan et al., 2021	HIV susceptible individuals between 15 and 64 years of age in the United States of America	HIV	16 evidence based interventions that prevent and target, diagnose and treat HIV.	Intervention costs	QALYs<br><br>ICER (USD per QALY)	Absolute (between group variance)/ relative (Theil index) and index of disparity
